# Climate and Competitive Status Modulate the Variation in Secondary Metabolites More in Leaves Than in Fine Roots of *Betula pendula*

**DOI:** 10.3389/fpls.2021.746165

**Published:** 2021-11-25

**Authors:** Arvo Tullus, Linda Rusalepp, Reimo Lutter, Katrin Rosenvald, Ants Kaasik, Lars Rytter, Sari Kontunen-Soppela, Elina Oksanen

**Affiliations:** ^1^Institute of Ecology and Earth Sciences, University of Tartu, Tartu, Estonia; ^2^Institute of Veterinary Medicine and Animal Sciences, Estonian University of Life Sciences, Tartu, Estonia; ^3^Institute of Forestry and Rural Engineering, Estonian University of Life Sciences, Tartu, Estonia; ^4^Rytter Science, Röstånga, Sweden; ^5^Department of Environmental and Biological Sciences, University of Eastern Finland, Joensuu, Finland

**Keywords:** climate, intraspecific competition, Fennoscandia, fine roots, leaf chemistry, plant phenolics, silver birch

## Abstract

Plant secondary metabolites have many important functions; they also determine the productivity and resilience of trees under climate change. The effects of environmental factors on secondary metabolites are much better understood in above-ground than in below-ground part of the tree. Competition is a crucial biotic stress factor, but little is known about the interaction effect of climate and competition on the secondary chemistry of trees. Moreover, competition effect is usually overlooked when analyzing the sources of variation in the secondary chemistry. Our aim was to clarify the effects of competitive status, within-crown light environment, and climate on the secondary chemistry of silver birch (*Betula pendula* Roth). We sampled leaves (from upper and lower crown) and fine roots from competitively dominant and suppressed *B. pendula* trees in plantations along a latitudinal gradient (56–67° N) in Fennoscandia, with mean annual temperature (MAT) range: −1 to 8°C. Secondary metabolites in leaves (SM_L_) and fine roots (SM_FR_) were determined with an HPLC-qTOF mass spectrometer. We found that SM_L_ content increased significantly with MAT. The effect of competitive stress on SM_L_ strengthened in colder climates (MAT<4°C). Competition and shade initiated a few similar responses in SM_L_. SM_FR_ varied less with MAT. Suppressed trees allocated relatively more resources to SM_L_ in warmer climates and to SM_FR_ in colder ones. Our study revealed that the content and profile of secondary metabolites (mostly phenolic defense compounds and growth regulators) in leaves of *B. pendula* varied with climate and reflected the trees’ defense requirements against herbivory, exposure to irradiance, and competitive status (resource supply). The metabolic profile of fine roots reflected, besides defense requirements, also different below-ground competition strategies in warmer and colder climates. An increase in carbon assimilation to secondary compounds can be expected at northern latitudes due to climate change.

## Introduction

Plants’ ability to cope with global changes and withstand environmental stresses is strongly related to their production and accumulation of secondary metabolites. For example, phenolic compounds (e.g., flavonoids) offer the primary layer of defense against herbivores, microbial pathogens, and UV radiation but also contribute to numerous other plant–environment interactions, including allelopathy and symbiosis (Bennett and Wallsgrove, 1994; Witzell and Martín, 2008; Chen et al., 2009; Eyles et al., 2010; Pierik et al., 2013). Some secondary compounds, such as jasmonates, besides their defensive role also act as growth regulators and inhibit plant growth in response to unfavorable conditions (Huang et al., 2017). Recently, the role of secondary metabolites as antioxidants has been shown to be greater than previously thought (Close and McArthur, 2002; Agati et al., 2012, 2020). The general ecological and evolutionary trends in plant secondary chemistry are explained by several theories, including the carbon/nutrient balance (CNB) hypothesis (Bryant et al., 1983) and the growth–differentiation balance (GDB) hypothesis (Herms and Mattson, 1992). However, these theories are often debated (Hamilton et al., 2001; Close and McArthur, 2002; Massad et al., 2014) and do not always reflect the responses of different metabolic pathways and individual compounds (Koricheva et al., 1998; Keinänen et al., 1999). In the case of surplus carbon (C) fixed in photosynthesis, which due to nutrient limitation is not expendable for growth, some of it is allocated to the synthesis of secondary C-based compounds (Prescott et al., 2020). Thus, the content of secondary metabolites can reflect the overall stress level of plants, including trees (Lihavainen et al., 2016; Gargallo-Garriga et al., 2018). At the same time, in some environments or plant developmental stages, more resources can be invested in secondary metabolism also without a direct cost on growth (Stamp, 2004; Massad et al., 2014).

Neighborhood competition for limited environmental resources is among the most crucial biotic stress factors; it affects the growth, functioning, and architecture of trees as well as the structural development of forests (Coomes and Grubb, 2000; Forrester, 2019). Although several studies have revealed that tree growth responses to global change drivers are significantly mediated by competition (McDonald et al., 2002; Tullus et al., 2017; Maes et al., 2019), the effect of competition on the secondary metabolism of trees has rarely been investigated in local (Donaldson et al., 2006; Rivoal et al., 2011) or latitudinal (clinal) studies (Poeydebat et al., 2021) addressing growth–defense partitioning of trees in various environments. Studies with trees suggest that the importance of competition (among other environmental growth factors) increases in more productive environments, whereas intensity of competition is less variable along ecological gradients (Kunstler et al., 2011; Laurent et al., 2017). At the same time, the roles of competition for light and soil resources can be contrasting in plants (Jucker et al., 2014). Above-ground light competition does not only occur just between individual trees but also within the tree crown. In shaded leaves, photosynthesis is limited by light availability and hence there is also less C available for the production of defensive compounds (Henriksson et al., 2003), although intra-tree allocation can depend on other factors as well, e.g., the shade tolerance of the species (Naidu et al., 1998). At higher latitudes of Northern Europe, tree growth is more N-limited (Lebauer and Treseder, 2008; Högberg et al., 2021; Possen et al., 2021) and less controlled by water availability (Bergh et al., 1999). In line with the optimal biomass partitioning (OBP) theory (more is allocated to an organ, which acquires the limiting resource), trees growing in the cold/harsh climates of high latitudes/altitudes have a relatively higher root mass fraction (Luo et al., 2012; Tenkanen et al., 2021) and could be more strongly restricted by below-ground competition for the scarce nutrients from neighboring plants (Berendse and Möller, 2009) as well as from soil microbial communities (Thébault et al., 2014). Following the optimal defense (OD) hypothesis (the value of a plant tissue is determined by the reduction in plant fitness resulting from the loss of that tissue (McKey, 1974)), one may assume that roots are more valuable in the north. Despite their vital role, the secondary chemistry of tree fine roots has been less studied although it is associated with ectomycorrhizal activity (Rosinger et al., 2020) and root exudation (Bertin et al., 2007) and thus clearly contributes to overall root competition. Phenolics in plant roots can vary with biotic and abiotic stress conditions (Saviranta et al., 2010), and they have a role in root defense (Lanoue et al., 2010) as well as in modulating soil microbial communities (Zwetsloot et al., 2020).

Several studies have shown that herbivory increases toward lower (warmer) latitudes (Lim et al., 2015; Moreira et al., 2018; Zvereva et al., 2020; Poeydebat et al., 2021). In accordance with the latitudinal herbivory-defense (LHD) hypothesis (Johnson and Rasmann, 2011), plants’ defensive chemistry should also follow the same pattern (Poeydebat et al., 2021), whereas high herbivore pressure favors constitutive over induced defense (Anstett et al., 2016; Bixenmann et al., 2016). However, latitudinal tests of the mentioned relationships including other potential modifiers of resource availability, e.g., plant density (Comita et al., 2014) and neighborhood diversity (Castagneyrol et al., 2018), are still scarce, especially from northern latitudes. Moreover, the negative latitude–herbivory relationship is not consistently proven and opposite observations exist as well (Adams et al., 2009; Moles et al., 2011).

Silver birch (*Betula pendula* Roth), as a fast-growing light-demanding early successional tree species, exhibits a strong shade avoidance syndrome in response to competing neighbors (Gilbert et al., 2001). This in turn may affect growth/defense partitioning in individual trees when the birch stand reaches competitive stage as plants tend to favor growth over defense under such circumstances (Fernández-Milmanda et al., 2020). *Betula pendula* is a widely distributed and economically important deciduous tree in Northern Europe (Hynynen et al., 2010; Dubois et al., 2020). The metabolic profile of *B. pendula* is known to vary with genotype and latitude of origin across Northern Europe (Deepak et al., 2018), but the effect of competition has rarely been clarified. Vegetation dynamics models predict that the distribution range of *B. pendula* will likely shift considerably northward as a consequence of climate change (Dyderski et al., 2018).

Our aim was to clarify the effects of a tree’s competitive status in a local neighborhood, within-crown light environment, and climate on the secondary chemistry of *B. pendula* in monospecific plantations after canopy closure and onset of competitive growth stage. Based on data from six *B. pendula* plantations along a latitudinal gradient (56–67° N) in Fennoscandia, we analyzed the secondary metabolites in leaves (SM_L_) and fine roots (SM_FR_) to detect whether and to what extent the responses to competitive status and climate vary between the two vital organs. We hypothesized that: (i) Both SM_L_ and SM_FR_ decrease northward with latitude; (ii) shade from neighboring trees and from lower canopy position initiates similar responses in leaf metabolic profile; and (iii) the relative allocation of C-based secondary metabolites in roots vs. leaves is higher in suppressed trees and at northern latitudes.

## Materials and Methods

### Studied Sites

**Figure 1:**
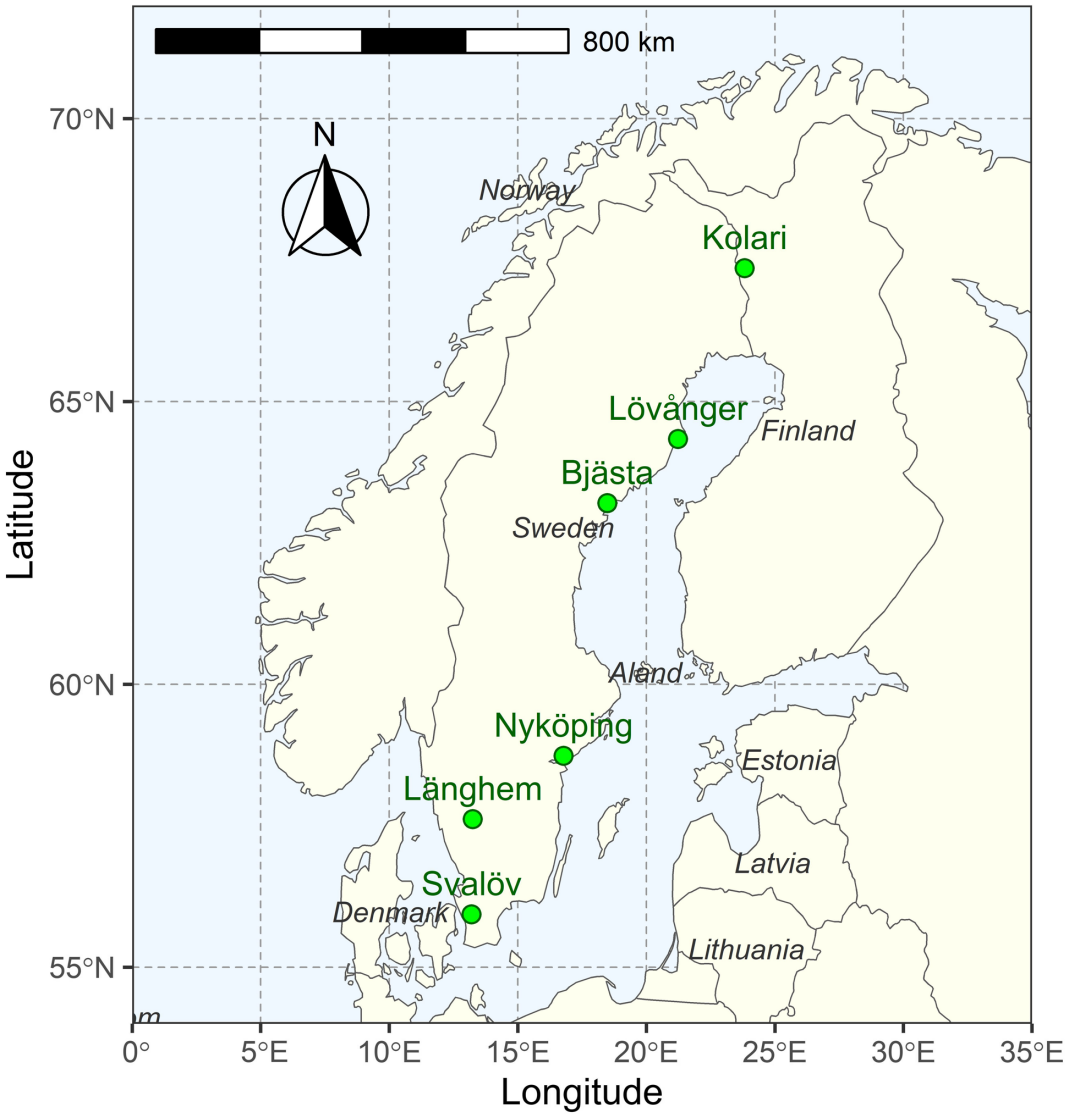
Locations of the study sites in Northern Europe.

**Figure 2:**
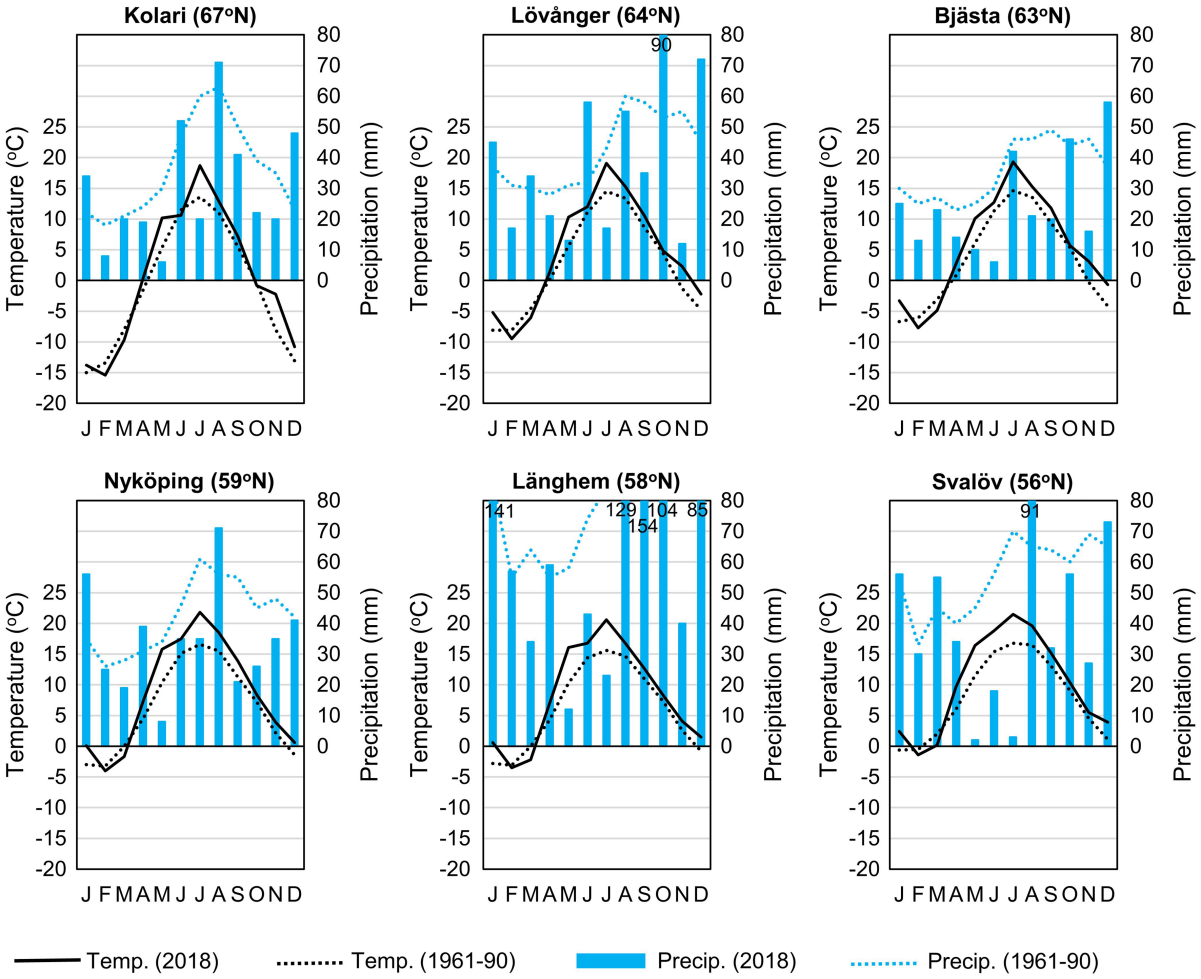
Climate diagrams of the study sites (in sampling year 2018 and long term; SMHI, 2018).

This study was conducted in young (6–9-year-old) silver birch (*Betula pendula* Roth) plantations that had reached canopy closure and entered an intensive competitive growth phase. The six study sites were distributed along a 1,400-km latitudinal gradient (56–67° N) in Northern Europe (Fennoscandia), where long-term mean annual temperature (MAT) ranges from −1 to 8°C (Figures 1, 2; Table 1).

**Table 1:** Study site and plantation characteristics.

Plantation	Geographic coordinates		Climate characteristics^a^		Topsoil (0–30cm) characteristics^b^			*Betula pendula* plantations characteristics^c^		
	Latitude	Longitude	MAT (°C)	Prec (mm)	pH	C/N	Texture class	Previous landuse	Plant material (year of planting)	Stand density (stems ha^−1^)
Svalöv (SE)	55°56’	13°12’	7.9	666	5.2	12.0	Silty clay	Grain, fallow	Seed orchard Ekebo-4 (2009)	1,600
Länghem (SE)	57°37’	13°15’	6.1	975	5.6	14.1	Loam	Grain, willow	Seed orchard Ekebo-4 (2009)	1,600
Nyköping (SE)	58°44’	16°47’	6.3	507	5.3	11.4	Clay loam	Grain, fallow	Seed orchard Ekebo-4 (2012)	1,600
Bjästa (SE)	63°12’	18°29’	3.4	428	5.3	15.7	Silt loam	Arable crops	Finnish seed orchard, FP431 (2009)	1,600
Lövånger (SE)	64°20’	21°14’	2.6	503	5.5	15.6	Silt loam	Grain, fallow	Finnish seed orchard, SV413 (2009)	1,600
Kolari (FI)	67°21’	23°50’	−1.0	432	5.2	-	Sandy till	Arable crops	Mixture of genotypes from southern, central and northern Finland (2010)	3,000

a *SMHI (2018); MAT, long-term (1961–1990) mean annual temperature; Prec., long-term (1961–1990) mean annual precipitation*.

b *Swedish (SE) sites – Rytter (2012), Finnish (FI) site – Heimonen et al. (2015)*.

c *Swedish (SE) sites – Rytter and Rytter (2020)*.

### Leaf and Root Sampling From Model Trees

**Figure 3:**
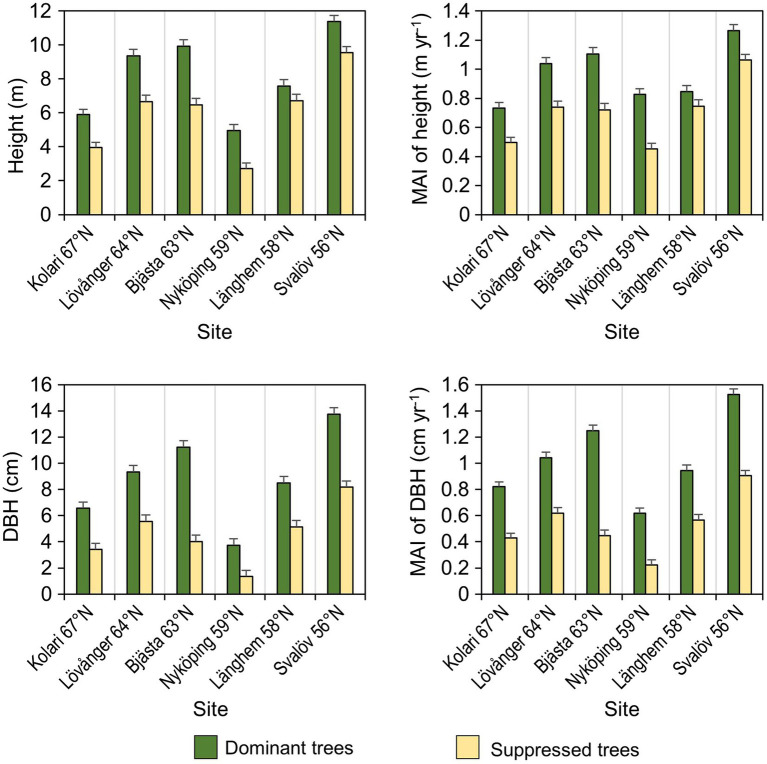
Mean (±SE) growth characteristics (DBH, diameter of the stem at breast height; MAI, mean annual increment) of the sample trees (*n*=60).

At each site, five pairs of trees were sampled in July 2018 and their basic growth characteristics were measured (Figure 3). Each pair included a competitively dominant and a competitively suppressed tree neighbor; the distance between the two individuals varied from 1.2 to 3.5m. The model trees were chosen so that the height of the dominant tree exceeded the average height of its closest neighboring trees by at least 1m and the height of the suppressed tree was at least 1m less than the average height of its closest neighbors. From each model tree (10 trees from each site, 60 trees in total), two composite leaf samples and one composite root sample were collected.

The leaves were collected from the upper and lower quarters of the south-facing side of the living crown. From both crown layers, a branch was cut with a 9.6-m extendable pole cutter (Ergo-Schnitt, Germany) and 35–40 mature leaves without visible damage (to avoid local damage-induced defense) were collected. Fresh leaf blade area was measured with the software package WinFolia (Regent Instruments Canada Inc.). Thereafter, the leaves were dried to constant weight at 60°C in a desiccator and weighed to the nearest 0.01g to estimate the mean specific leaf area (SLA).

Three to four 10–20cm root fragments were extracted near to each model tree stem (it was checked visually that the root initiated from the given stem) from 0 to 20cm topsoil layer and preserved in a portable cooler during transportation to the laboratory. Fine roots (diameter<2mm) were separated in the laboratory from each root sample and dried to constant weight at 40°C for further analysis. The plant material samples were ground prior to the chemical analysis.

### Foliar Nutrients

Total nitrogen content ([N], %) was analyzed with the Kjeldahl method, using a Kjeltec Auto 1030 Analyzer (Foss Tecator AB, Höganäs, Sweden). Phosphorus ([P], %) was determined spectrophotometrically *via* Kjeldahl digestion, using a FIAstar 5000 Analyzer (FOSS Tecator AB), and potassium ([K], %) was determined by flame photometry using the Sherwood Model 425 Flame Photometer in the Laboratory of Plant Biochemistry at the Estonian University of Life Sciences.

### LC–MS Analysis

The leaf and fine root extracts were chromatographically analyzed using a 1290 Infinity system (Agilent Technologies, Waldbronn, Germany) coupled to an Agilent 6450 Q-TOF mass spectrometer equipped with a Jetstream ESI source.

We tested 80, 50, and 20% (v/v) methanol solutions in water for extracting ground fine roots and leaves, of which the 50% methanol solution gave the highest chromatographic peaks and was therefore chosen as the extraction solvent. The ground plant material was extracted (1:20w/v) for 24h in darkness at room temperature, with occasional shaking. Thereafter, the extracts were centrifuged at 4,000rpm for 15min with an Eppendorf 5810 R centrifuge (Eppendorf AG, Hamburg, Germany). The obtained supernatants were filtered through a Minisart® RC 15mm, 0.45μm non-sterile syringe filter (Sartorius AG, Göttingen, Germany). The filtrate was subjected to a Zorbax 300SB-C18 column (2.1mm×150mm; 5μm; Agilent Technologies) kept at 40°C. For elution of the samples, a gradient of 0.1% formic acid in water (A) and acetonitrile (B) was used as follows: 0min 5% B, 31.8min 32% B, 32.8–40.0min 99% B, 40.1min 5% B, regeneration time 7min. The eluent flow rate was set to 0.3mlmin^−1^, and the injection size was 2.5μl. The mass spectrometer was working in negative ionization mode in the mass-to-charge ratio (*m/z*) range of 100–1,700amu. Data acquisition and initial data processing were carried out using MassHunter software (Agilent Technologies).

Compounds were identified by comparison of the *m/z* value, retention time, UV spectra, and MS^2^ fragmentation patterns with standards or by comparison with data from the literature or the METLIN database (Agilent Technologies).

### Statistical Analysis

As the first step, we reduced the dimensionality of the original dataset by assigning each secondary metabolite compound to one of the following groups: diaryl compounds (DAR), flavonoids (FLA), hydroxycinnamates (HC), hydroxybenzoic acids (HBA), jasmonates (JA), monoaryl compounds (MAR), sesquiterpenoids (ST), terpene glycosides (TG). Thereafter, the extracted ion chromatogram peak areas of secondary metabolites were standardized and group means were calculated. Variation in the group means of standardized contents was analyzed with linear mixed models (LMM), which were fitted separately for leaf and fine root data. In the LMMs, the error structure accounted for spatial dependencies in the data. Sample plot, tree pair, and tree (only for leaf data) were added as random factors. The fixed factors were competitive status (dominant or suppressed) and when analyzing SM_L_ also canopy position (upper or lower). Long-term mean annual temperature (MAT) was used as the main explanatory variable describing the climate differences along the latitudinal gradient. MAT also describes the length of the vegetation period for these sites (linear regression, *R*^2^=0.99, *p*<0.001). The average height of the dominant trees sampled at each site was added as a covariate to capture confounding variation arising from differences in soil properties and sample tree age. LMMs with afore-mentioned design were also used to explain the variation in leaf morphological traits and macronutrient contents. Additionally, the effects of competitive status and MAT on the SM_FR_/SM_L_ ratio (using average SM_L_ of the two canopy positions) were tested with LMM including sample plot and tree pair as random factors. The models included the main effects and second-order interactions of all explanatory variables.

The LMM analysis was performed with the function *lmer* in the package *lme4* of R Statistics software (R Core Team, 2021). Model predictions and *post hoc* comparisons between group means and trends were made with the package *emmeans*. When a significant interaction effect of competitive status and MAT was detected, pairwise comparisons of the competitive status class means predicted for each degree across the studied MAT range (−1 to 8°C) were made to clarify at what temperatures the differences between dominant and suppressed trees were significant. Linear correlation matrices were calculated to characterize general relationships among the secondary metabolite contents and tree and site characteristics.

Normality of model residuals was checked from residual histograms and Q–Q plots. When necessary, *log*- or square-root transformation was applied. A significance level of *α*=0.05 was used to reject the null hypothesis after statistical tests.

## Results

### Foliar Secondary Metabolites

Altogether, 68 SM_L_ compounds were identified (Supplementary Figure 1; Supplementary Table 1), the most represented groups of compounds being flavonoids (FLA; *n*=31) and hydroxycinnamates (HC; *n*=15) followed by sesquiterpenoids (ST; *n*=5), jasmonates (JA; *n*=5), monoaryl compounds (MAR; *n*=5), terpene glycosides (TG; *n*=4), and hydroxybenzoic acids (HBA; *n*=3). Most of the identified SM_L_ are associated with the shikimate–phenylpropanoid pathway. Coumaric and caffeic acid derivatives made up the HC group. The FLA group was represented by numerous quercetin glycosides, of which some malonyl- and acetylglycosides only occurred at certain study sites. Also, the ST compound roseoside 2 was only present in about half of the samples and it was not related to the study site.

**Figure 4:**
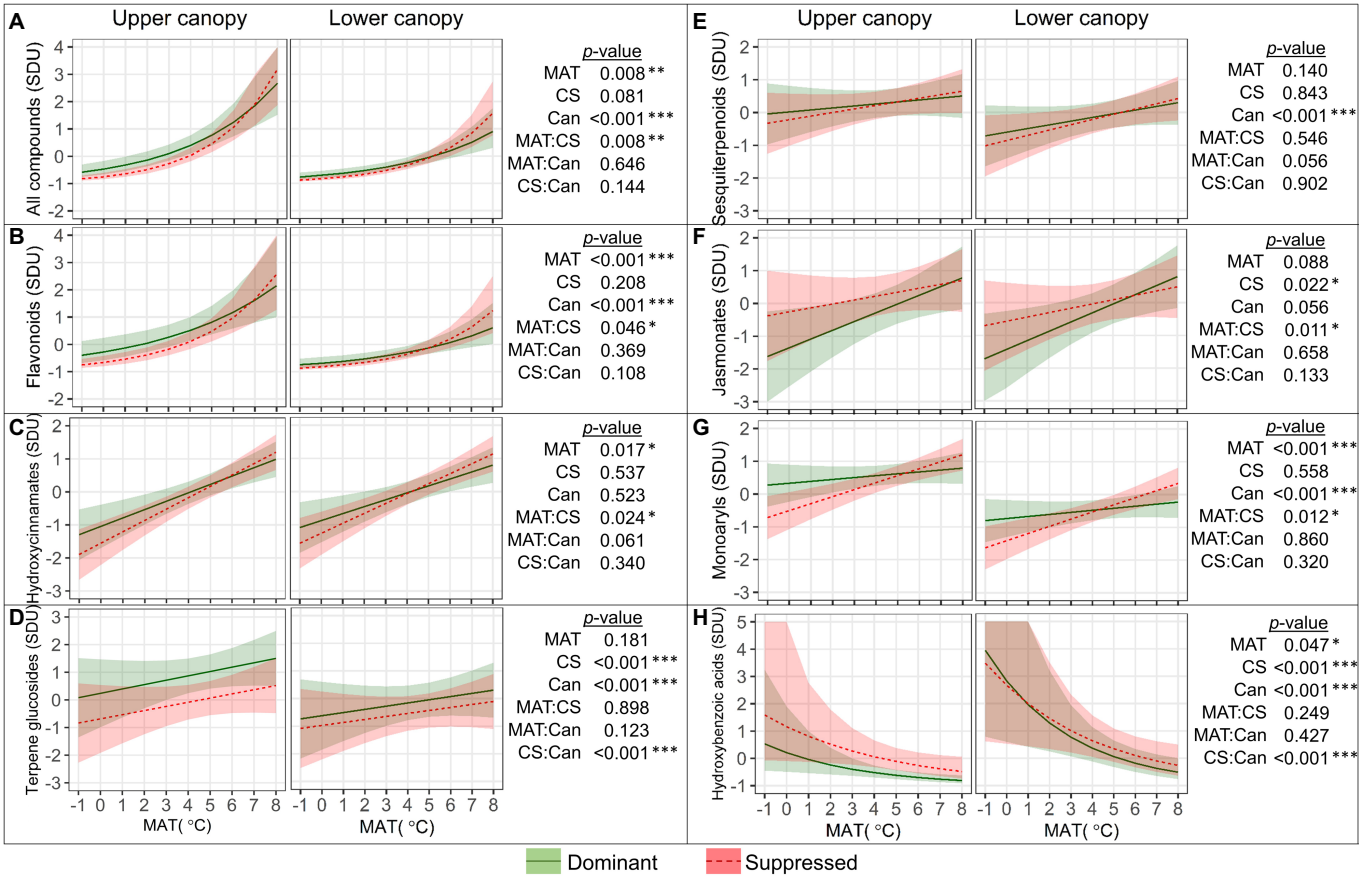
Relationships between the quantity (SDU, standard deviation units) of all foliar secondary metabolite compounds **(A)** and groups of compounds **(B–H)** with mean annual temperature (MAT) in the upper and lower canopy of competitively dominant and suppressed silver birches (predicted mean trend with 95% confidence bands based on LMM). Curvilinear trendlines indicate that data were *log*-transformed prior to LMM analysis and the model predictions were back-transformed for data visualization. The significance of the factors (MAT; CS, competitive status; Can, canopy position) is shown next to the graphs: *p<0.05, **p<0.01, ***p<0.001.

Significant main and second-order interactive effects of MAT, canopy position (Can), and a tree’s competitive status (CS) on SM_L_ groups were detected (Figure 4). The mean standardized SM_L_ content (Figure 4A) increased with MAT, and the increase was significantly greater (“MAT×CS,” *p*=0.008) in suppressed trees (by 0.46 SD per degree within the MAT range from −1 to 8°C) than in dominant trees (by 0.37 SD per degree). Accordingly, the difference in SM_L_ between dominant and suppressed trees became more distinct in colder climates (MAT<4°C) but was not revealed at warmer latitudes. The mean SM_L_ content was significantly affected by canopy position (*p*<0.001) throughout the studied MAT range, and it was 0.54 SD greater in upper compared to lower canopy leaves (Figure 4A).

The quantity of the most numerous groups of compounds (FLA and HC) was greater in dominant trees than in suppressed trees in colder climates, but as their content increased with MAT more steeply in suppressed trees (“MAT×CS,” *p*<0.05), the effect of competitive status became non-significant in warmer climates (Figures 4B,C). The FLA content (Figure 4B) was greater in upper canopy leaves across the MAT range (*p*<0.001). The TG content (Figure 4D) was higher in upper canopy leaves, and the effect of canopy position was more pronounced in dominant trees (“CS×Can,” *p*<0.001). The ST content (Figure 4E) was higher in upper canopy leaves (*p*<0.001) but did not vary with MAT or competitive status. The JA content (Figure 4F) was significantly lower in leaves of dominant trees in colder climates; however, as it increased significantly with MAT contrarily to that in suppressed trees, this distinction did not appear in warm climates (“MAT×CS,” *p*=0.011). The MAR content (Figure 4G) was significantly higher in leaves of dominant trees in colder climates, but it did not increase with MAT contrarily to that in suppressed trees (“MAT×CS,” *p*=0.012), and consequently, no difference between the competitive status classes was observed in warmer climates. The MAR content was significantly higher in upper canopy leaves than in lower canopy leaves (*p*<0.001). HBA (Figure 4H) were the only group of SM_L_ that decreased with MAT (by −0.29 SD per degree, *p*=0.047) and their content was greater in lower canopy leaves, whereas competitive status effect (greater content in suppressed trees) was revealed only in upper canopy leaves (“CS×Can,” *p*<0.001).

### Leaf Morphological Traits and Macronutrients

**Figure 5:**
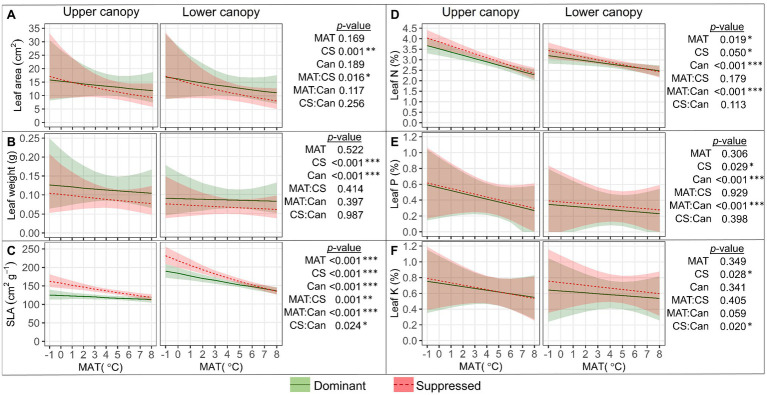
Relationships between leaf size characteristics (SLA, specific leaf area; **A–C**), leaf macronutrient content **(D–F)**, and mean annual temperature (MAT) in upper and lower canopy of competitively dominant and suppressed silver birches (predicted mean trend with 95% confidence bands based on LMM). Curvilinear trendlines indicate that data were *log*-transformed prior to LMM analysis and the model predictions were back-transformed for data visualization. The significance of the factors (MAT; CS, competitive status; Can, canopy position) is shown next to the graphs: *p<0.05, **p<0.01, ***p<0.001.

Single leaf blade area (Figure 5A) decreased with MAT, and the decrease was steeper in suppressed trees than in dominant trees (“MAT×CS,” *p*=0.016); as a result, the leaf area of dominant trees exceeded that of suppressed trees only in warmer climates. Leaves of dominant trees (*p*<0.001) and from the upper canopy position (*p*<0.001) were significantly heavier than leaves of suppressed trees and from the lower canopy (Figure 5B). SLA (Figure 5C) decreased with MAT, and this trend was stronger in suppressed trees (“MAT×CS,” *p*=0.001) and in lower canopy positions (“MAT×Can,” *p*<0.001). In colder climates, the difference in SLA between dominant and suppressed trees as well as that between the upper and lower canopy was considerably greater. Leaf [N] and [P] (Figures 5D,E) decreased with MAT, more markedly in the upper canopy (“MAT×Can,” *p*<0.001), and the content of both nutrients was slightly higher in suppressed trees (*p*<0.05). Leaf [K] (Figure 5F) was significantly higher in the lower canopy of suppressed trees than dominant trees (“CS×Can,” *p*=0.02).

Pairwise correlations (based on pooled data) showed several significant relationships between SM_L_ groups, leaf nutrients, and morphological traits (Table 2). In addition, sampling height was often a stronger predictor of SM_L_ than the total height of the sample tree. Pairwise correlations between SM_L_ and MAT accorded with the outcomes of LMM analyses.

**Table 2:** Linear correlations between the secondary metabolite contents in leaves, sample tree characteristics and mean annual temperature.

Group of compounds	Tree characteristics								MAT
	*H*_tree_	*H*_sample_	LA	LW	SLA	[N]	[P]	[K]	
JA	**0.33**	**0.29**	−0.03	0.16	**−0.26**	−0.07	−0.12	0.06	**0.59**
FLA	−0.03	**0.28**	**−0.43**	0.04	**−0.61**	**−0.65**	−0.17	**−0.32**	**0.60**
HC	**−0.24**	−0.12	**−0.54**	**−0.21**	**−0.42**	**−0.77**	**−0.38**	**−0.37**	**0.59**
ST	−0.10	0.13	**−0.29**	−0.01	**−0.41**	**−0.21**	0.00	**−0.23**	**0.30**
*log*TG	0.08	**0.40**	**−0.28**	**0.26**	**−0.71**	**−0.44**	−0.08	**−0.24**	**0.42**
*log*HBA	**−0.28**	**−0.49**	**0.22**	**−0.31**	**0.74**	**0.44**	0.08	0.16	**−0.70**
MAR	**0.28**	**0.61**	−0.14	**0.31**	**−0.63**	−0.16	0.09	−0.02	**0.44**
*log*SUM	−0.05	**0.24**	**−0.49**	0.01	**−0.66**	**−0.69**	**−0.24**	**−0.34**	**0.69**

*JA, jasmonates; FLA, flavonoids; HC, hydroxycinnamates; ST, sesquiterpenoids; TG, terpene glycosides; HBA, hydroxybenzoic acids; MAR, monoaryl compounds; SUM, sum of all compounds; sample tree characteristics (H_tree_, height of the sample tree; H_sample_, sampling height), concentration of foliar nutrients (N, P, K), leaf blade area (LA), leaf blade weight (LW), specific leaf area (SLA), and mean annual temperature (MAT). **p<0.05**; **p#x003C;0.001***.

### Fine Root Secondary Metabolites

Altogether, 39 SM_FR_ were identified (Supplementary Figure 2; Supplementary Table 2), the most numerous groups of compounds being diarylheptanoids (DAR; *n*=12) and FLA (*n*=11) followed by HBA (*n*=7), MAR (arylbutanoids; *n*=6), JA (*n*=2) and HC (*n*=1). The most abundant compound in fine roots was the arylbutanoid rhamnosyl(epi)rhododendrin. The FLA group contained catechin, proanthocyanidin dimers and trimers, and hydroxyflavanone *C*-glycosides. Platyphyllonol rhamnoside was the most abundant compound in the DAR group. Syringic and vanillic acid derivatives made up the HBA group.

**Figure 6:**
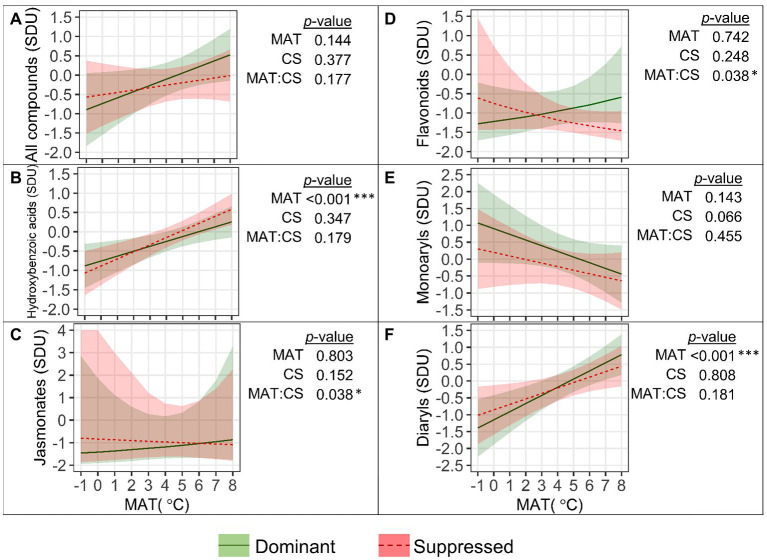
Relationships between the quantity (SDU, standard deviation units) of all fine root secondary metabolite compounds **(A)** and groups of compounds **(B–F)** with mean annual temperature (MAT) in competitively dominant and suppressed silver birches (predicted mean trend with 95% confidence bands based on LMM). Curvilinear trendlines indicate that data were *log*-transformed prior to LMM analysis and the model predictions were back-transformed for data visualization. The significance of the factors (MAT; CS, competitive status) is shown next to the graphs: *p<0.05, ***p<0.001.

The mean standardized content of SM_FR_ (Figure 6A) was not significantly affected by MAT, while some trends occurred in different groups of compounds (Figures 6B–F). The content of HBA (Figure 6B) and DAR (Figure 6F) in fine roots increased significantly with MAT, by 0.16 and 0.20 (resp.) SD per degree (both: *p*<0.001). The JA content (Figure 6C) was higher in the fine roots of suppressed trees in colder climates with a MAT<4°C (“MAT×CS,” *p*=0.038). The competitive status effect on the FLA content in the fine roots (Figure 6D) was revealed only in warmer climates with a MAT>6°C (“MAT×CS,” *p*=0.038). Among FLA, for catechins the “MAT×CS” interactive effect was especially strong (*p*<0.001). Catechins in fine roots decreased with MAT, and their content in the fine roots of suppressed trees was significantly higher in colder climates and significantly lower in warmer climates, compared to dominant trees. The content of arylbutanoids in fine roots was not affected by the MAT or competitive status of the tree (Figure 6E).

Pairwise correlations (based on pooled data) showed several significant relationships between SM_FR_ groups, sample tree height and MAT (Table 3). Pairwise correlations between SM_FR_ and MAT were generally in accordance with the outcomes of LMM analyses, although there was a weak significant correlation between MAT and the total content of SM_FR_ (*p*=0.044), which was not supported by LMM (*p*=0.144). Sample tree height was a somewhat stronger predictor of SM_FR_ than SM_L_.

**Table 3:** Linear correlations between secondary metabolite content in fine roots, sample tree height and mean annual temperature.

Group of compounds	*H*_tree_	MAT
JA	**0.34**	0.20
FLA	**0.26**	0.00
HBA	**0.57**	**0.43**
MAR	−0.05	**−0.44**
DAR	**0.36**	**0.48**
SUM	**0.42**	**0.26**

*JA, jasmonates; FLA, flavonoids; HBA, hydroxybenzoic acids; MAR, monoaryl compounds; DAR, diaryl compounds; SUM, sum of all compounds; sample tree height (H_tree_), and mean annual temperature (MAT). **p<0.05**; **p<0.001***.

**Figure 7:**
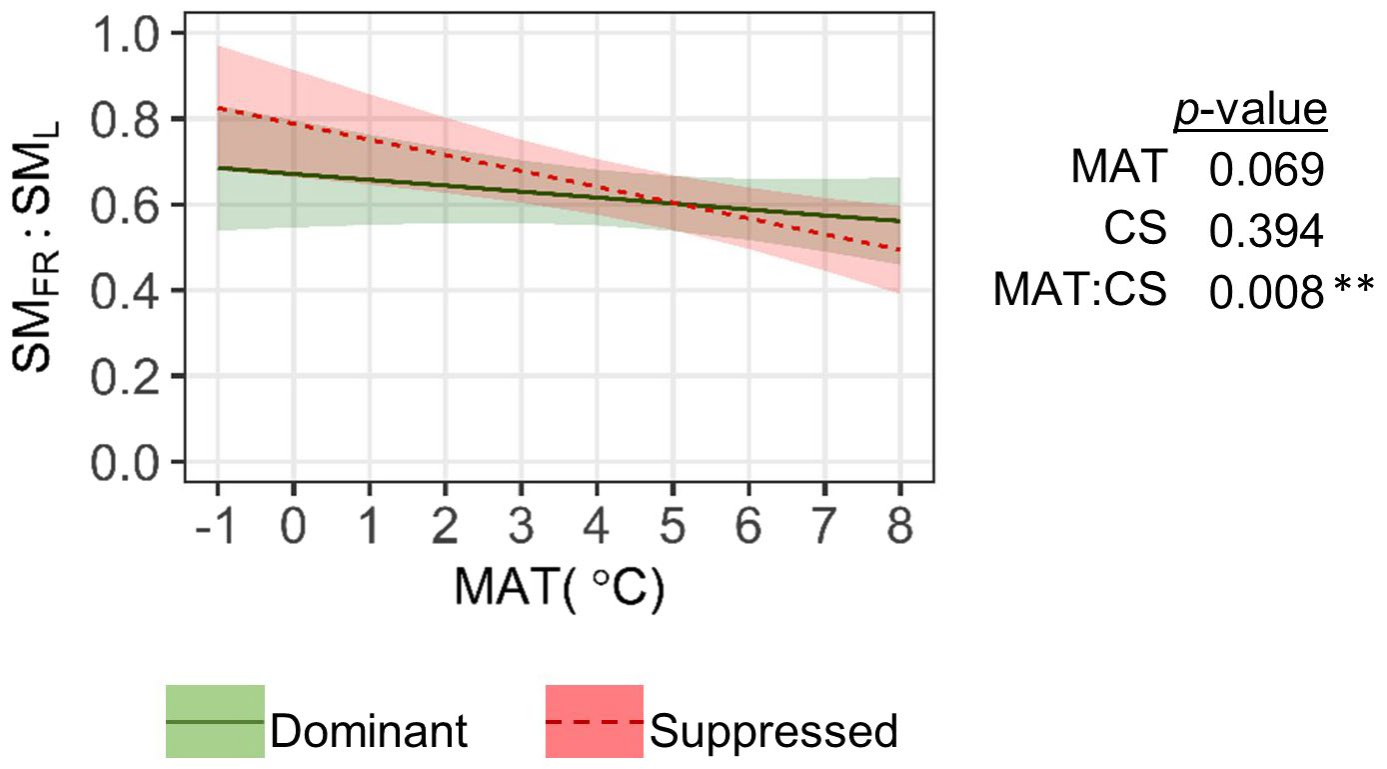
Relationship between mean annual temperature (MAT) and the ratio of total content (sum of EIC peak areas) of secondary metabolites in fine roots (SM_FR_) to leaves (SM_L_). Leaf data for each sample tree were averaged across two canopy positions. The significance of the factors (MAT; CS, competitive status) according to LMM is shown next to the graph: **p<0.01.

We detected a significant pattern in allocation between SM_FR_ and SM_L_ based on their estimated total content (Figure 7). In dominant trees, the respective ratio was 0.61±0.02 and varied little with MAT. In competitively suppressed trees, the ratio decreased considerably with MAT from 0.83 at the coldest to 0.49 at the warmest end of our latitudinal gradient. The SM_FR_/SM_L_ ratio became significantly (*p*<0.05) higher in suppressed trees than in dominant trees when MAT fell below 3°C.

## Discussion

Our latitudinal study showed that MAT and intraspecific competition both affected significantly the SM_L_ and SM_FR_ profiles of *B. pendula*. Both factors had a stronger effect on SM_L_ than on SM_FR_. We found evidence supporting our research hypotheses: Both SM_L_ and SM_FR_ decreased northward with latitude; shade from over-topping neighbors and from lower canopy position initiated some similar responses in SM_L_; and the relative allocation of C-based secondary metabolites in roots vs. leaves increased with latitude, especially in competitively suppressed trees.

As expected, in warmer temperate/oceanic climates with a longer growing period, both dominant and suppressed *B. pendula* trees increased their C investment into secondary (defense) compounds. This may partly be explained by their exposure to higher levels of leaf herbivory in warmer and more resourceful conditions (Forister et al., 2015; Poeydebat et al., 2021). Herbivory of *B. pendula* decreases with latitude in Northern Europe, and our study year (2018) was no exception from this pattern (Zvereva et al., 2020). Hence, our results support the LHD hypothesis (Johnson and Rasmann, 2011). A lower content of chemical defense compounds is likely one reason behind the higher palatability of birch genotypes of northern origin and of lower growth rate also seen in Finnish common garden experiments (Heimonen et al., 2015, 2017). A greater need to protect against more intensive irradiance during the longer growing period in the southern sites could be another reason for the observed increase in SM_L_ with MAT (Close and McArthur, 2002). Besides the total content of SM_L_, such a trend was observed in FLA which are known for their antioxidative role in plants (Agati et al., 2012, 2020). Thus, the observed increase in SM_L_ toward the equator was probably shaped by both clinal changes in herbivory as well as irradiance.

The total SM_L_ content was lower in competitively suppressed trees than in dominant trees, especially in the colder boreal/sub-arctic climates with a short growing period. Obviously, when suppressed by neighbors, *B. pendula* as an early successional shade-intolerant species allocates resources to growth at the expense of defense in stands which have recently reached competitive growth stage. A similar outcome has been observed in another early successional species, *Pinus halepensis* (Rivoal et al., 2011). Besides, shaded trees need less protection against photo-damage, which is one of the main functions of SM_L_. We hypothesized that both within-crown light environment and competitive status have similar effects on SM_L_ caused by shading. If so, then differences in dominant vs. suppressed and upper vs. lower canopy position should be in the same direction. This was observed only in two groups of compounds (TG and HBA). The main effect of canopy position on SM_L_ was observed more frequently in SM_L_ groups, irrespective of competitive status. The production of glycosylated precursors of volatile compounds, including ST and TG, was related to canopy position and/or sampling height but not considerably to MAT. Also, previous studies have indicated variable relationships between birch provenance and VOC emissions, where higher emissions have been found at both warmer (Hartikainen et al., 2012) and colder sites (Maja et al., 2015), indicating the complexity of the environmental factors affecting volatile compounds and yearly fluctuations. Hence, shading due to overtopping neighbors as well as due to the lower canopy position could trigger some similar responses in leaf metabolic profile, although this was not an universal response in all groups of SM_L_ compounds. Apparently, the involved physiological mechanisms behind the responses to shade and competition may differ, which requires further investigation.

Our results imply that individual compounds and pathways do not exclusively behave in a similar manner to the total summary content of secondary metabolites, which is consistent with findings in other studies (Koricheva et al., 1998; Keinänen et al., 1999). For example, in contrast to total SM_L_, JA and HBA in leaves were higher in suppressed trees and HBA decreased with MAT. The HBA compound group, containing protocatechuic and gallic acid derivatives, has similarities to hydrolysable tannins, and in accordance with results of Adams et al. (2009), the HBA content decreases with MAT. The JA content was higher in suppressed trees in colder climates. JA act as growth inhibitors when a plant is exposed to stresses, which enables the plant to allocate more resources to defense. This has previously often been recorded in herbaceous plants under various biotic and abiotic stresses (Huang et al., 2017), but rarely in trees under competitive stress (Rusalepp et al., 2021).

The differences in performance between the upper and lower canopy leaves reflect the morphological and functional acclimation to microclimate and light environment. We found that SLA increased toward lower canopy parts, while [N] and leaf dry weight decreased, which agrees with previous studies with *B. pendula* across a shorter latitudinal gradient (Deepak et al., 2019). The current study showed that leaf [N] decreased more with shade in colder climates. Leaf morphology (SLA in particular) is strongly affected by the light environment, which has high annual variation at high latitudes (cold climates; Poorter et al., 2019). Although the lower solar angle at higher latitudes is almost fully compensated by longer days during summer months, SLA clearly decreases with increasing daily light integral (DLI; Poorter et al., 2019). In their review, Poorter et al. (2019) concluded that light availability is a dominant factor in plant competition. In particular, photosynthetically active leaf area and area-based photosynthetic capacity (indicated by SLA) have great ecological and competitive value. We found that individual leaf blade area and SLA decreased with MAT, especially in suppressed trees, while individual leaf weight was related to canopy position and a tree’s competitive status. High SLA indicates high light capture by leaf tissue mass, whereas low SLA reflects mass allocation that increases longevity, robustness, and defense (Westoby, 1998). Hence, in colder climates trees minimize the construction cost of leaf tissues, especially in the presence of (additional) competitive stress. A negative relationship between the main SM_L_ (FLA and HC) and leaf blade area, leaf weight, and SLA indicates that a warmer climate enhances SM_L_ production but favors smaller leaves with lower SLA. Similarly, previous studies with Finnish birches showed a negative correlation of FLA content with leaf area and SLA (Deepak et al., 2018). The decreased concentration of birch surface FLA with leaf expansion may be due to a dilution effect (Valkama et al., 2004). On the other hand, the magnitude of the MAT effect was greater for SM_L_ than for leaf area, also suggesting causes other than dilution. Mäenpää et al. (2011) earlier demonstrated that instead of increasing leaf size, *B. pendula* has a strategy to produce more leaves in warmer conditions to enhance canopy biomass. This is in agreement with our findings at individual leaf level although we did not determine the total leaf number or area in the present study.

The above-ground growth rate of sampled birches was only slightly inferior in the northern sites compared to the southern ones (Figure 3). This agrees with the estimation of growth and productivity for the same plantations in Sweden (Rytter and Lutter, 2020). Despite obvious growth constraints in the north (lower MAT, shorter growing season, slower N mineralization), the leaf macronutrient content was higher in the north, suggesting that N availability was not the main growth limiting factor at these sites. It has been found that the northern provenances of *B. pendula* allocate more biomass into roots than shoots compared to the southern ones (Tenkanen et al., 2021), which might have facilitated N uptake. Increasing N content in *B. pendula* leaves with latitude agrees with findings for *Pinus sylvestris* (Reich et al., 1996; Oleksyn et al., 2002), suggesting that higher foliar N can partially compensate for the limitations on growth imposed by low N availability and temperature. Higher leaf N content observed in colder climates could reflect both decreased N dilution with slower growth and a physiological adaptation to low-temperature environments (Weih and Karlsson, 2001).

The CNB hypothesis and surplus C hypothesis (Prescott et al., 2020) both predict that carbohydrates accumulated in excess of growth requirements will be allocated to C-based secondary metabolism. Accordingly, we observed a negative relationship between leaf [N] and SM_L_. In this respect, N-limitation and surplus C were characteristic for southern sites. In summer 2018, Northern Europe was affected by a heatwave and drought may then have increased the production of some phenolics in tree leaves (Barchet et al., 2013). However, the chemistry of leaves and fine roots in July was apparently more shaped by conditions in the spring during their formation before the heatwave peaked.

In contrast to that of SM_L_, the total content of SM_FR_ was not significantly related to MAT or the competitive status of the tree. Since roots do not need protection against irradiance, their metabolic profile is apparently shaped by the need to defend against herbivory and other interactions with the below-ground environment. Root herbivory is generally much less studied than above-ground herbivory, but should follow a similar pattern, being higher in more fertile environments (Stevens and Jones, 2006). However, when analyzing separate groups of compounds, some significant effects of MAT and CS were detected. The HBA content in fine roots increased considerably with MAT. HBA represent low molecular weight organic acids, which when exudated into soil may stimulate microbial activity (Zwetsloot et al., 2020) and facilitate nutrient acquisition (Sokolova, 2020). This is somewhat surprising, as generally trees’ “microbial priming” effect is thought to be stronger in less fertile soils (Meier et al., 2020). Diaryl compounds in fine roots increased significantly with MAT, which may be associated with stronger herbivory defense, as, for instance, platyphylloside and its metabolite centrolobol reduce the digestibility of above-ground birch tissues (Sunnerheim et al., 1988). However, no relationship with root herbivory has previously been documented. The FLA catechin is considered to be a phytotoxic allelochemical (Treutter, 2006). In our gradient, it varied in fine roots with MAT, notably in suppressed trees, where it was lower in warmer climates and higher in colder ones compared to dominant trees. Catechin suppresses soil microbial processes (Zwetsloot et al., 2020) and may thus enable trees to secure nutrients in harsher conditions.

Although we did not measure the foliage and fine root biomass of the sample trees, we showed increasing allocation to SM_FR_ vs. SM_L_ per mass unit toward higher latitudes in suppressed trees. This might reflect greater importance of below-ground processes including competition in low-resource environments (Berendse and Möller, 2009). Accordingly, in colder climates, *B. pendula* has a larger relative root biomass fraction (Tenkanen et al., 2021). Fine root turnover is slower in the north (Yuan and Chen, 2010; Finér et al., 2011), and longer-living roots may need more defense compounds to ensure longevity, especially in the case of suppressed trees which also experience greater above-ground resource deficiency. Leaf life span is longer at lower latitudes, which could be another reason for the observed higher concentration of SM_L_ and lower ratio of SM_FR_ to SM_L_ at lower latitudes.

Tree secondary chemistry is, among other factors, shaped by age (developmental stage; Smith et al., 2011; Couture et al., 2014). However, age of *B. pendula* in this study was relatively invariant and we have considered this in statistical data analysis by using dominant trees’ average height as a proxy for possible age- and site-related confounding effects. Various genotypes of *B. pendula* were growing in the six plantations (Table 1), but as none of them was represented in all sites, we could not test the genotype × environment effect. Soil fertility (Keinänen et al., 1999) and soil type (Kolstad et al., 2016) can affect tree secondary chemistry. All the sites were former agricultural lands where soil texture and main chemical properties did not vary considerably (Table 1). Hence, the observed significant trends in SM_L_ and SM_FR_ with MAT along the 1,400km gradient were revealed across locally adapted as well as geographically shifted genotypes but under relatively uniform soil conditions. However, it cannot be excluded that some of the observed variation in secondary chemistry of *B. pendula* was genotypic (Keinänen et al., 1999; Deepak et al., 2018).

## Conclusion

We found that in young *B. pendula* trees, SM_L_ increased significantly with MAT (ranging from −1 to 8°C along the 1,400km latitudinal gradient [56–67 °N]), supporting the latitudinal herbivory-defense hypothesis and concurring with the latitudinal variation in irradiance intensity.

Differences in SM_L_ between competitively dominant and suppressed trees reflected the trees’ requirements for protection against irradiance as well as their resource supply. Generally, *B. pendula* allocated more resources for defense in upper canopy leaves. More shaded environments in the lower tree crown and under competitive stress resulted only in a few similar effects on SM_L_. The effect of competitive status on SM_L_ became distinct in colder climates (MAT<4°C) and was not observed at lower latitudes, suggesting that in harsher conditions, *B. pendula* as a shade-intolerant early successional species prefers shade avoidance over defense.

The SM_FR_ content varied less with MAT, but also indicated a higher level of anti-herbivory defense in warmer climates. In accordance with the optimal defense hypothesis, competitively stressed *B. pendula* trees allocated relatively more resources to leaf defense in warmer climates and to root defense in colder ones.

The findings of this study suggest that *B. pendula* trees growing at higher latitudes and under competitive stress may be more vulnerable to pathogens and herbivory, especially if climate warming continues. Therefore, an increase in carbon assimilation to secondary compounds can be expected under northern conditions as a consequence of climate change.

As secondary metabolites of dominant trees were less affected by MAT compared to suppressed trees, stand density regulation could be a silvicultural option to mitigate the mentioned climate change hazard, although this requires further investigation including other stand age classes, site types, and tree species, as well as the consideration of annual fluctuations.

## Data Availability Statement

The raw data supporting the conclusions of this article will be made available by the authors, without undue reservation.

## Author Contributions

AT conceived the idea. All authors helped to design the study. AT, RL, and LRy conducted the fieldwork. LRy provided background data for the sites in Sweden. EO and SK-S provided background data for the site in Finland. KR supervised the collection and preparation of fine root samples. LRu performed the HPLC analysis. AK and AT performed statistical data analysis. AT and LRu wrote the first draft of the manuscript. All authors contributed to the article and approved the submitted version.

## Funding

This work was supported by the Estonian Research Council (grants PSG7 to AT and PSG600 to RL) of the Estonian Ministry of Education and Research.

## Conflict of Interest

The authors declare that the research was conducted in the absence of any commercial or financial relationships that could be construed as a potential conflict of interest.

## Publisher’s Note

All claims expressed in this article are solely those of the authors and do not necessarily represent those of their affiliated organizations, or those of the publisher, the editors and the reviewers. Any product that may be evaluated in this article, or claim that may be made by its manufacturer, is not guaranteed or endorsed by the publisher.

## Abbreviations

Can: Canopy position
CS: Competitive status
DAR: Diaryl compounds
FLA: Flavonoids
HBA: Hydroxybenzoic acids
HC: Hydroxycinnamates
JA: Jasmonates
LMM: Linear mixed models
MAR: Monoaryl compounds
MAT: Mean annual temperature
SDU: Standard deviation units
SM_FR_: Secondary metabolites in fine roots
SM_L_: Secondary metabolites in leaves
ST: Sesquiterpenoids
TG: Terpene glycosides
