# Supplementary material for: Climate and Competitive Status Modulate the Variation in Secondary Metabolites More in Leaves Than in Fine Roots of Betula pendula
Source: Front Plant Sci. 2021 Nov 25;12:746165. doi: 10.3389/fpls.2021.746165 (PMC8655902; doi:10.3389/fpls.2021.746165)
Supplement: Supplementary file 1 [file Data_Sheet_1.docx]

Supplementary Material

# Supplementary Figures


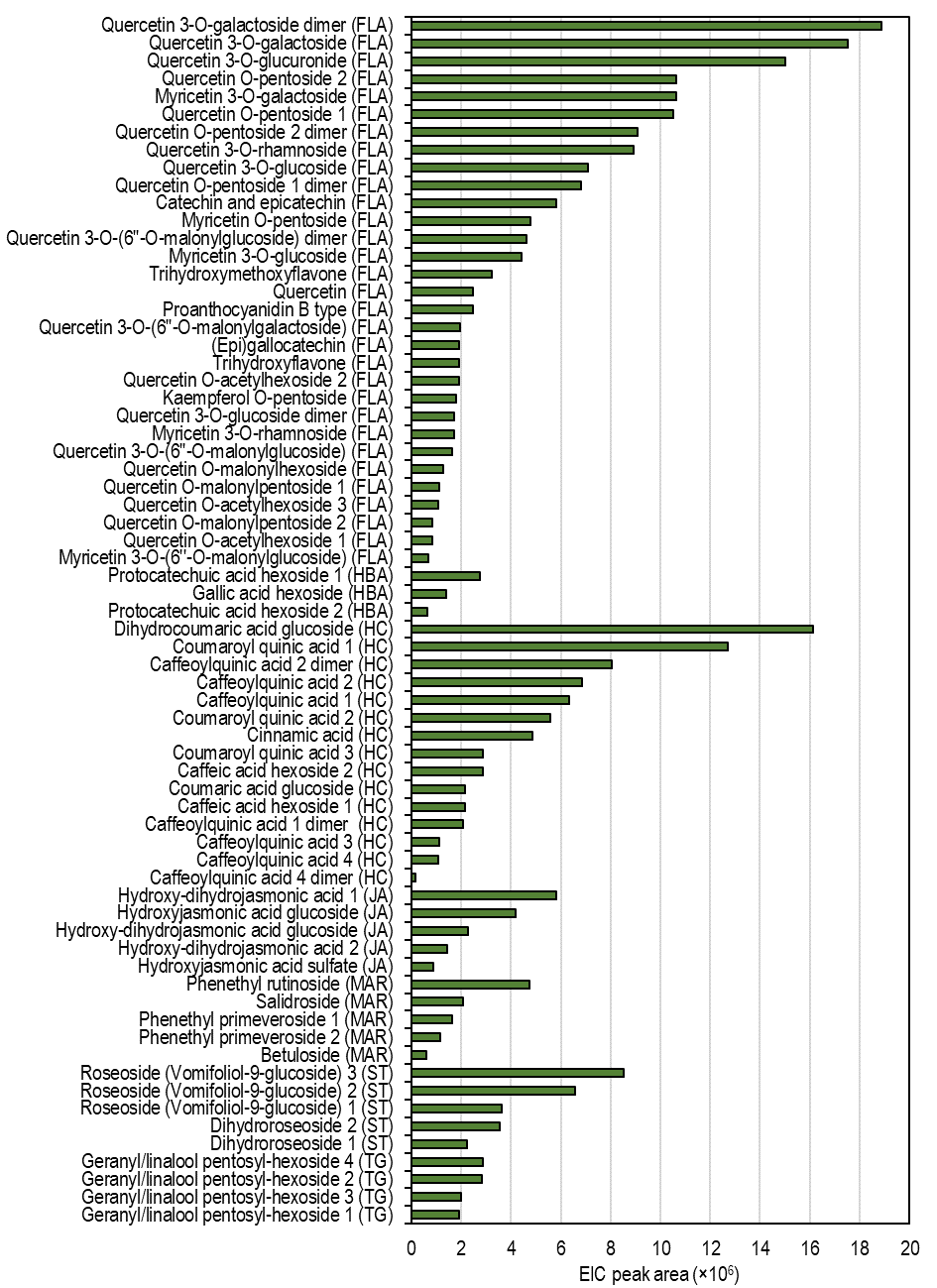


**Supplementary Figure 1.** Average content of secondary metabolites (*n* = 68) identified in leaves of *B. pendula*, based on pooled data across sites and tree size and canopy position groups. Abbreviations in brackets indicate the class of compounds (JA, jasmonates; FLA, flavonoids; HC, hydroxycinnamates; ST, sesquiterpenoids; TG, terpene glycosides; HBA, hydroxybenzoic acids; MAR, monoaryl compounds).


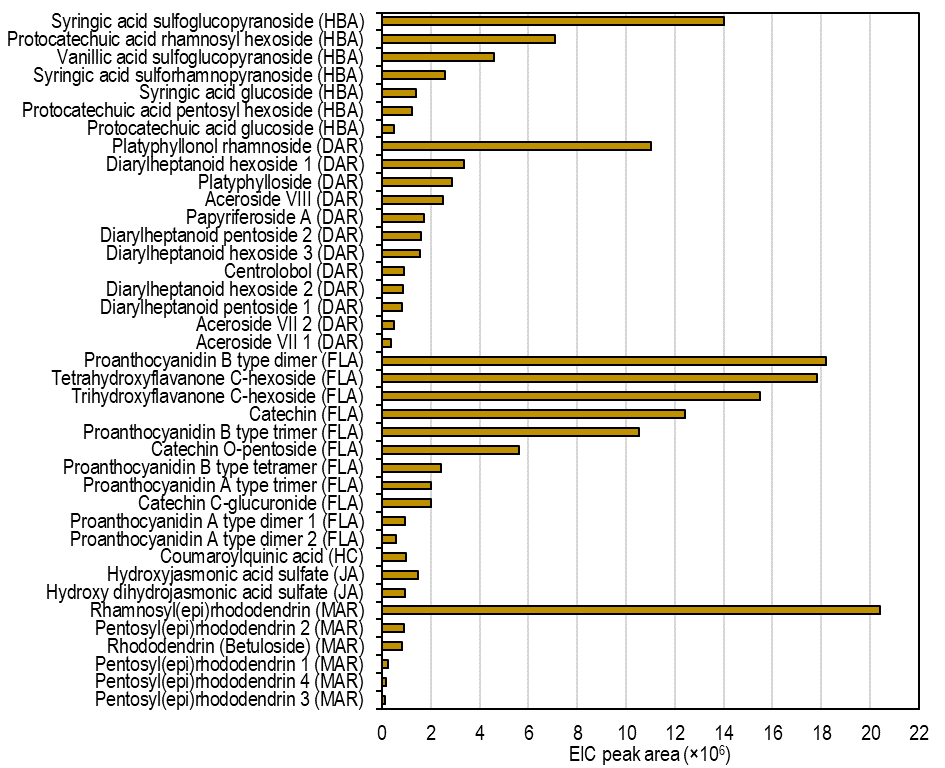


**Supplementary Figure 2.** Average content of secondary metabolites (*n* = 39) identified in fine roots of *B. pendula*, based on pooled data across sites and tree size groups. Abbreviations in brackets indicate the class of compounds (DAR, diaryl compounds; JA, jasmonates; FLA, flavonoids; HC, hydroxycinnamates; HBA, hydroxybenzoic acids; MAR, monoaryl compounds).

# Supplementary Tables

**Supplementary Table 2.** Retention times (*t_R_*), mass-to-charge ratios (*m/z*), mass differences, ion type, molecular formula and compound class of metabolites detected in the methanolic extracts of leaves of *Betula pendula*.

| Compound | *t_R_* (min) | Monoisotopic *m/z* | Observed *m/z* | Mass difference (ppm) | Molecular formula | Ion type | Compound class |
| --- | --- | --- | --- | --- | --- | --- | --- |
| Trihydroxyflavone | 26.5 | 269.0455 | 269.0452 | −1.1 | C_15_H_10_O_5_ | [M-H]^−^ | FLA |
| Catechin and epicatechin | 5.5 | 289.0718 | 289.0707 | −3.8 | C_15_H_14_O_6_ | [M-H]^−^ | FLA |
| Trihydroxymethoxyflavone | 27.4 | 299.0561 | 299.0562 | 0.3 | C_16_H_12_O_6_ | [M-H]^−^ | FLA |
| Quercetin | 22.4 | 301.0354 | 301.0347 | −2.3 | C_15_H_10_O_7_ | [M-H]^−^ | FLA |
| (Epi)gallocatechin | 2.5 | 305.0667 | 305.0658 | −3.0 | C_15_H_14_O_7_ | [M-H]^−^ | FLA |
| Kaempferol *O*-pentoside | 19.5 | 417.0827 | 417.0829 | 0.5 | C_20_H_18_O_10_ | [M-H]^−^ | FLA |
| Quercetin *O*-pentoside 1 | 16.5 | 433.0776 | 433.0764 | −2.8 | C_20_H_18_O_11_ | [M-H]^−^ | FLA |
| Quercetin *O*-pentoside 2 | 17.0 | 433.0776 | 433.0790 | 3.2 | C_20_H_18_O_11_ | [M-H]^−^ | FLA |
| Quercetin 3-*O*-rhamnoside | 17.7 | 447.0933 | 447.0948 | 3.4 | C_21_H_20_O_11_ | [M-H]^−^ | FLA |
| Myricetin *O*-pentoside | 14.2 | 449.0725 | 449.0744 | 4.2 | C_20_H_18_O_12_ | [M-H]^−^ | FLA |
| Myricetin 3-*O*-rhamnoside | 14.4 | 463.0882 | 463.0876 | −1.3 | C_21_H_20_O_12_ | [M-H]^−^ | FLA |
| Quercetin 3-*O*-galactoside | 14.8 | 463.0882 | 463.0861 | −4.5 | C_21_H_20_O_12_ | [M-H]^−^ | FLA |
| Quercetin 3-*O*-glucoside | 15.4 | 463.0882 | 463.0863 | −4.1 | C_21_H_20_O_12_ | [M-H]^−^ | FLA |
| Quercetin 3-*O*-glucuronide | 15.1 | 477.0675 | 477.0690 | 3.1 | C_21_H_18_O_13_ | [M-H]^−^ | FLA |
| Myricetin 3-*O*-galactoside | 12.3 | 479.0831 | 479.0849 | 3.8 | C_21_H_20_O_13_ | [M-H]^−^ | FLA |
| Myricetin 3-*O*-glucoside | 12.7 | 479.0831 | 479.0834 | 0.6 | C_21_H_20_O_13_ | [M-H]^−^ | FLA |
| Quercetin *O*-acetylhexoside 1 | 16.3 | 505.0988 | 505.0967 | −4.2 | C_23_H_22_O_13_ | [M-H]^−^ | FLA |
| Quercetin *O*-acetylhexoside 2 | 16.8 | 505.0988 | 505.1008 | 4.0 | C_23_H_22_O_13_ | [M-H]^−^ | FLA |
| Quercetin *O*-acetylhexoside 3 | 18.2 | 505.0988 | 505.0972 | −3.2 | C_23_H_22_O_13_ | [M-H]^−^ | FLA |
| Quercetin *O-*malonylpentoside 1 | 18.8 | 519.0780 | 519.0759 | −4.0 | C_23_H_20_O_14_ | [M-H]^−^ | FLA |
| Quercetin *O*-malonylpentoside 2 | 19.6 | 519.0780 | 519.0779 | −0.2 | C_23_H_20_O_14_ | [M-H]^−^ | FLA |
| Quercetin 3-*O-*(6''-*O*-malonylgalactoside) | 16.3 | 549.0886 | 549.0868 | −3.3 | C_24_H_22_O_15_ | [M-H]^−^ | FLA |
| Quercetin 3-*O-*(6''-*O*-malonylglucoside) | 16.8 | 549.0886 | 549.0905 | 3.5 | C_24_H_22_O_15_ | [M-H]^−^ | FLA |
| Quercetin *O*-malonylhexoside | 18.2 | 549.0886 | 549.0868 | −3.3 | C_24_H_22_O_15_ | [M-H]^−^ | FLA |
| Myricetin 3-*O*-(6''-*O*-malonylglucoside) | 14.3 | 565.0835 | 565.0819 | −2.8 | C_24_H_22_O_16_ | [M-H]^−^ | FLA |
| Proanthocyanidin B type dimer | 4.9 | 577.1351 | 577.1334 | −2.9 | C_30_H_26_O_12_ | [M-H]^−^ | FLA |
| Quercetin *O*-pentoside 1 dimer | 16.5 | 867.1625 | 867.1661 | 4.2 | C_40_H_36_O_22_ | [2M-H]^−^ | FLA |
| Quercetin *O*-pentoside 2 dimer | 17.0 | 867.1625 | 867.1595 | −3.5 | C_40_H_36_O_22_ | [2M-H]^−^ | FLA |
| Quercetin 3-*O-*galactoside dimer | 14.8 | 927.1837 | 927.1794 | −4.6 | C_42_H_40_O_24_ | [2M-H]^−^ | FLA |
| Quercetin 3-*O*-glucoside dimer | 15.4 | 927.1837 | 927.1874 | 4.0 | C_42_H_40_O_24_ | [2M-H]^−^ | FLA |
| Quercetin 3-*O*-(6''-*O*-malonylglucoside) dimer | 16.8 | 1099.1845 | 1099.1800 | −4.1 | C_48_H_44_O_30_ | [2M-H]^−^ | FLA |
| Protocatechuic acid hexoside 1 | 2.3 | 315.0722 | 315.0728 | 1.9 | C_13_H_16_O_9_ | [M-H]^−^ | HBA |
| Protocatechuic acid hexoside 2 | 3.0 | 315.0722 | 315.0728 | 1.9 | C_13_H_16_O_9_ | [M-H]^−^ | HBA |
| Gallic acid hexoside | 2.0 | 331.0671 | 331.0676 | 1.5 | C_13_H_16_O_10_ | [M-H]^−^ | HBA |
| Cinnamic acid | 5.1 | 147.0452 | 147.0451 | −0.7 | C_9_H_8_O_2_ | [M-H]^−^ | HC |
| Coumaric acid glucoside | 6.1 | 325.0929 | 325.0928 | −0.3 | C_15_H_18_O_8_ | [M-H]^−^ | HC |
| Dihydrocoumaric acid glucoside | 5.1 | 327.1085 | 327.1087 | 0.6 | C_15_H_20_O_8_ | [M-H]^−^ | HC |
| Coumaroyl quinic acid 1 | 5.0 | 337.0929 | 337.0923 | −1.8 | C_16_H_18_O_8_ | [M-H]^−^ | HC |
| Coumaroyl quinic acid 2 | 9.0 | 337.0929 | 337.0924 | −1.5 | C_16_H_18_O_8_ | [M-H]^−^ | HC |
| Coumaroyl quinic acid 3 | 11.1 | 337.0929 | 337.0922 | −2.1 | C_16_H_18_O_8_ | [M-H]^−^ | HC |
| Caffeic acid hexoside 1 | 4.1 | 341.0878 | 341.0881 | 0.9 | C_15_H_18_O_9_ | [M-H]^−^ | HC |
| Caffeic acid hexoside 2 | 4.6 | 341.0878 | 341.0883 | 1.5 | C_15_H_18_O_9_ | [M-H]^−^ | HC |
| Caffeoylquinic acid 1 | 3.3 | 353.0878 | 353.0880 | 0.6 | C_16_H_18_O_9_ | [M-H]^−^ | HC |
| Caffeoylquinic acid 2 | 6.3 | 353.0878 | 353.0885 | 2.0 | C_16_H_18_O_9_ | [M-H]^−^ | HC |
| Caffeoylquinic acid 3 | 6.8 | 353.0878 | 353.0886 | 2.3 | C_16_H_18_O_9_ | [M-H]^−^ | HC |
| Caffeoylquinic acid 4 | 8.5 | 353.0878 | 353.0876 | −0.6 | C_16_H_18_O_9_ | [M-H]^−^ | HC |
| Caffeoylquinic acid 1 dimer | 3.3 | 707.1829 | 707.1830 | 0.1 | C_16_H_18_O_9_ | [2M-H]^−^ | HC |
| Caffeoylquinic acid 2 dimer | 6.3 | 707.1829 | 707.1814 | −2.1 | C_16_H_18_O_9_ | [2M-H]^−^ | HC |
| Caffeoylquinic acid 4 dimer | 8.5 | 707.1829 | 707.1821 | −1.1 | C_16_H_18_O_9_ | [2M-H]^−^ | HC |
| Hydroxy-dihydrojasmonic acid 1 | 29.7 | 227.1289 | 227.1289 | 0.0 | C_12_H_20_O_4_ | [M-H]^−^ | JA |
| Hydroxy-dihydrojasmonic acid 2 | 31.1 | 227.1289 | 227.1289 | 0.0 | C_12_H_20_O_4_ | [M-H]^−^ | JA |
| Hydroxyjasmonic acid sulfate | 7.7 | 305.0700 | 305.0705 | 1.6 | C_12_H_18_O_7_S | [M-H]^−^ | JA |
| Hydroxyjasmonic acid glucoside | 9.3 | 387.1661 | 387.1650 | −2.8 | C_18_H_28_O_9_ | [M-H]^−^ | JA |
| Hydroxy-dihydrojasmonic acid glucoside | 10.5 | 389.1817 | 389.1817 | 0.0 | C_18_H_30_O_9_ | [M-H]^−^ | JA |
| Salidroside | 4.0 | 345.1191 | 345.1192 | 0.3 | C_14_H_20_O_7_ | [M+HCOO]^−^ | MAR |
| Betuloside | 10.6 | 373.1504 | 373.1498 | −1.6 | C_16_H_24_O_7_ | [M-H]^−^ | MAR |
| Phenethyl rutinoside | 20.2 | 429.1766 | 429.1757 | −2.1 | C_20_H_30_O_10_ | [M-H]^−^ | MAR |
| Phenethyl primeveroside 1 | 10.1 | 461.1664 | 461.1661 | −0.7 | C_19_H_28_O_10_ | [M+HCOO]^−^ | MAR |
| Phenethyl primeveroside 2 | 11.2 | 461.1664 | 461.1662 | −0.4 | C_19_H_28_O_10_ | [M+HCOO]^−^ | MAR |
| Roseoside (Vomifoliol glucoside) 1 | 8.4 | 431.1923 | 431.1904 | −4.4 | C_19_H_30_O_8_ | [M-H]^−^ | ST |
| Roseoside (Vomifoliol glucoside) 2 | 8.9 | 431.1923 | 431.1904 | −4.4 | C_19_H_30_O_8_ | [M-H]^−^ | ST |
| Roseoside (Vomifoliol glucoside) 3 | 9.1 | 431.1923 | 431.1904 | −4.4 | C_19_H_30_O_8_ | [M-H]^−^ | ST |
| Dihydroroseoside 1 | 10.6 | 433.2079 | 433.2072 | −1.6 | C_19_H_32_O_8_ | [M-H]^−^ | ST |
| Dihydroroseoside 2 | 11.4 | 433.2079 | 433.2083 | 0.9 | C_19_H_32_O_8_ | [M-H]^−^ | ST |
| Geranyl/linalool pentosyl-hexoside 1 | 23.5 | 493.2291 | 493.2283 | −1.6 | C_21_H_36_O_10_ | [M+HCOO]^−^ | TG |
| Geranyl/linalool pentosyl-hexoside 2 | 24.3 | 493.2291 | 493.2285 | −1.2 | C_21_H_36_O_10_ | [M+HCOO]^−^ | TG |
| Geranyl/linalool pentosyl-hexoside 3 | 24.7 | 493.2291 | 493.2280 | −2.2 | C_21_H_36_O_10_ | [M+HCOO]^−^ | TG |
| Geranyl/linalool pentosyl-hexoside 4 | 25.3 | 493.2291 | 493.2280 | −2.2 | C_21_H_36_O_10_ | [M+HCOO]^−^ | TG |

**Supplementary Table 2.** Retention times (*t_R_*), mass-to-charge ratios (*m/z*), mass differences, ion type, molecular formula and compound class of metabolites detected in the methanolic extracts of fine roots of *Betula pendula*.

| Compound | *t_R_* (min) | Monoisotopic *m/z* | Observed *m/z* | Mass difference (ppm) | Molecular formula | Ion type | Compound class |
| --- | --- | --- | --- | --- | --- | --- | --- |
| Protocatechuic acid glucoside | 2.3 | 315.0722 | 315.0714 | −2.5 | C_13_H_16_O_9_ | [M-H]^−^ | BA |
| Syringic acid glucoside | 3.4 | 359.0984 | 359.0989 | 1.4 | C_15_H_20_O_10_ | [M-H]^−^ | BA |
| Vanillic acid sulfoglucopyranoside | 2.1 | 409.0446 | 409.0434 | −2.9 | C_14_H_18_O_12_S | [M-H]^−^ | BA |
| Syringic acid sulforhamnopyranoside | 10.6 | 423.0603 | 423.0589 | −3.3 | C_15_H_20_O_12_S | [M-H]^−^ | BA |
| Syringic acid sulfoglucopyranoside | 2.6 | 439.0552 | 439.0537 | −3.4 | C_15_H_20_O_13_S | [M-H]^−^ | BA |
| Protocatechuic acid pentosyl hexoside | 3.2 | 447.1144 | 447.1127 | −3.8 | C_18_H_24_O_13_ | [M-H]^−^ | BA |
| Protocatechuic acid rhamnosyl hexoside | 3.8 | 461.1301 | 461.1307 | 1.3 | C_19_H_26_O_13_ | [M-H]^−^ | BA |
| Centrolobol | 33.7 | 299.1653 | 299.1647 | −2.0 | C_19_H_24_O_3_ | [M-H]^−^ | DAR |
| Aceroside VII 1 | 26.2 | 461.2181 | 461.2174 | −1.5 | C_25_H_34_O_8_ | [M-H]^−^ | DAR |
| Aceroside VII 2 | 27.5 | 461.2181 | 461.2167 | −3.0 | C_25_H_34_O_8_ | [M-H]^−^ | DAR |
| Diarylheptanoid pentoside 1 | 21.6 | 493.2291 | 493.2279 | −2.4 | C_21_H_36_O_10_ | [M+HCOO]^−^ | DAR |
| Diarylheptanoid pentoside 2 | 22.8 | 493.2291 | 493.2286 | −1.0 | C_21_H_36_O_10_ | [M+HCOO]^−^ | DAR |
| Platyphyllonol rhamnoside | 23.8 | 505.2291 | 505.2292 | 0.2 | C_22_H_36_O_10_ | [M+HCOO]^−^ | DAR |
| Platyphylloside | 20.3 | 521.2028 | 521.2016 | −2.3 | C_25_H_32_O_9_ | [M+HCOO]^−^ | DAR |
| Diarylheptanoid hexoside 1 | 30.2 | 533.2028 | 533.2028 | 0.0 | C_26_H_32_O_9_ | [M+HCOO]^−^ | DAR |
| Diarylheptanoid hexoside 2 | 17.4 | 569.2240 | 569.2233 | −1.2 | C_26_H_36_O_11_ | [M+HCOO]^−^ | DAR |
| Diarylheptanoid hexoside 3 | 19.7 | 581.1876 | 581.1864 | −2.1 | C_26_H_32_O_12_ | [M+HCOO]^−^ | DAR |
| Aceroside VIII | 26.7 | 593.2604 | 593.2595 | −1.5 | C_30_H_42_O_12_ | [M-H]^−^ | DAR |
| Papyriferoside A | 20.0 | 607.2396 | 607.2376 | −3.3 | C_30_H_40_O_13_ | [M-H]^−^ | DAR |
| Catechin | 5.5 | 289.0718 | 289.0719 | 0.3 | C_15_H_14_O_6_ | [M-H]^−^ | FLA |
| Catechin *O*-pentoside | 5.3 | 421.1140 | 421.1144 | 0.9 | C_20_H_22_O_10_ | [M-H]^−^ | FLA |
| Trihydroxyflavanone *C*-hexoside | 12.6 | 433.1140 | 433.1127 | −3.0 | C_21_H_22_O_10_ | [M-H]^−^ | FLA |
| Tetrahydroxyflavanone *C*-hexoside | 6.4 | 449.1089 | 449.1087 | −0.4 | C_21_H_22_O_11_ | [M-H]^−^ | FLA |
| Catechin *C*-glucuronide | 3.7 | 465.1038 | 465.1041 | 0.6 | C_21_H_22_O_12_ | [M-H]^−^ | FLA |
| Proanthocyanidin A type dimer 1 | 2.3 | 575.1195 | 575.1195 | 0.0 | C_30_H_24_O_12_ | [M-H]^−^ | FLA |
| Proanthocyanidin A type dimer 2 | 3.0 | 575.1195 | 575.1199 | 0.7 | C_30_H_24_O_12_ | [M-H]^−^ | FLA |
| Proanthocyanidin B type dimer | 4.9 | 577.1351 | 577.1350 | −0.2 | C_30_H_26_O_12_ | [M-H]^−^ | FLA |
| Proanthocyanidin A type trimer | 3.1 | 863.1829 | 863.1830 | 0.1 | C_45_H_36_O_18_ | [M-H]^−^ | FLA |
| Proanthocyanidin B type trimer | 5.6 | 865.1985 | 865.1985 | 0.0 | C_45_H_38_O_18_ | [M-H]^−^ | FLA |
| Proanthocyanidin B type tetramer | 7.5 | 1153.2619 | 1153.2579 | −3.5 | C_60_H_50_O_24_ | [M-H]^−^ | FLA |
| Coumaroylquinic acid | 9.1 | 337.0929 | 337.0920 | −2.7 | C_16_H_18_O_8_ | [M-H]^−^ | HC |
| Hydroxyjasmonic acid sulfate | 7.7 | 305.0700 | 305.0696 | −1.3 | C_12_H_18_O_7_S | [M-H]^−^ | JA |
| Hydroxy dihydrojasmonic acid sulfate | 8.8 | 307.0857 | 307.0847 | −3.3 | C_12_H_20_O_7_S | [M-H]^−^ | JA |
| Rhododendrin (Betuloside) | 10.7 | 373.1504 | 373.1499 | −1.3 | C_16_H_24_O_7_ | [M+HCOO]^−^ | MAR |
| Pentosyl(epi)rhododendrin 1 | 19.3 | 505.1927 | 505.1918 | −1.8 | C_21_H_32_O_11_ | [M+HCOO]^−^ | MAR |
| Pentosyl(epi)rhododendrin 2 | 19.7 | 505.1927 | 505.1916 | −2.2 | C_21_H_32_O_11_ | [M+HCOO]^−^ | MAR |
| Pentosyl(epi)rhododendrin 3 | 20.2 | 505.1927 | 505.1915 | −2.4 | C_21_H_32_O_11_ | [M+HCOO]^−^ | MAR |
| Pentosyl(epi)rhododendrin 4 | 20.6 | 505.1927 | 505.1907 | −4.0 | C_21_H_32_O_11_ | [M+HCOO]^−^ | MAR |
| Rhamnosyl(epi)rhododendrin | 20.5 | 519.2083 | 519.2071 | −2.3 | C_22_H_34_O_11_ | [M+HCOO]^−^ | MAR |
